# Electrostatic-free piezoresponse force microscopy

**DOI:** 10.1038/srep41657

**Published:** 2017-01-31

**Authors:** Sungho Kim, Daehee Seol, Xiaoli Lu, Marin Alexe, Yunseok Kim

**Affiliations:** 1 School of Advanced Materials Science and Engineering, Sungkyunkwan University (SKKU), Suwon, 440-746, Republic of Korea; 2 The State Key Discipline Laboratory of Wide Band Gap Semiconductor Technology, Xidian University, Xi’an, Shaanxi 710071, China; 3 Department of Physics, University of Warwick, Coventry CV4 7AL, United Kingdom

## Abstract

Contact and non-contact based atomic force microscopy (AFM) approaches have been extensively utilized to explore various nanoscale surface properties. In most AFM-based measurements, a concurrent electrostatic effect between the AFM tip/cantilever and sample surface can occur. This electrostatic effect often hinders accurate measurements. Thus, it is very important to quantify as well as remove the impact of the electrostatic effect on AFM-based measurements. In this study, we examine the impact of the electrostatic effect on the electromechanical (EM) response in piezoresponse force microscopy as a model AFM mode. We quantitatively studied the effects of increasing the external electric field and reducing the spring constant of a cantilever. Further, we explored ways to minimize the electrostatic effect. The results provide broad guidelines for quantitatively analyzing the EM response as well as, eventually, for obtaining the electrostatic-free EM response. The conclusions can be applied to other AFM-based measurements that are subject to a strong electrostatic effect between the AFM tip/cantilever and sample surface, regardless of contact and non-contact modes.

Since the advent of atomic force microscopy (AFM), various surface properties have been explored by utilizing contact and non-contact AFM-based approaches on the nanoscale^1^,^2^,^3^,^4^. In most AFM-based measurements, a concurrent electrostatic effect occurs between the AFM tip/cantilever and the sample surface^5^,^6^. This electrostatic effect is attributed to the electrostatic force induced by the Coulomb interaction between separated charges^7^. Even though this electrostatic force can be utilized for exploring surface electrical properties in the field of electrostatic force microscopy and Kelvin probe force microscopy (KPFM), in many cases, the electrostatic effect hinders AFM-based measurements for accessing true material properties^5^,^6^,^8^,^9^,^10^. For instance, in non-contact based magnetic force microscopy (MFM), magnetic and electrostatic forces can simultaneously affect MFM, distorting the measured response^11^. Furthermore, it is well known that the electrostatic effect can also affect the measured electromechanical (EM) response in contact-based dynamic force microscopy such as piezoresponse force microscopy (PFM) and electrochemical strain microscopy^5^,^12^,^13^. In particular, previously reported theoretical results prove that electrostrictive and electrostatic effects can always exist in voltage modulated AFM measurements^14^ and Hong, as well as Balke *et al*., estimated the impact of the electrostatic effect on the EM hysteresis loop measurements by comparing on- and off-field EM hysteresis loops^5^,^15^. Although the electrostatic effect has been widely studied, its impact is mainly examined when DC voltage is applied, *i.e*. on-field state. That is, as-grown electrostatic effect caused by the measured sample and its corresponding quantitative effect on the measured EM response have not received sufficient attention. For quantitatively analyzing the EM response as well as eventually accessing for the electrostatic-free EM response of the measured sample, it is necessary to recognize and quantify the contribution of the sample dependent electrostatic effect to the EM response.

In this paper, we investigate the impact of the electrostatic effect on the EM response and suggest a couple of different ways for the electrostatic-free PFM. As a model AFM system, we employed PFM and explored the impact of the electrostatic effect on the PFM response using PFM and KPFM in two model samples, a (001)-oriented epitaxial Pb(Zr_0.2_Ti_0.8_)O_3_ (PZT) thin film and periodically poled lithium niobate (PPLN). The as-grown surface potential induced electrostatic effect and its significant impact on the PFM images were observed in the PPLN. Consistent with previous reports, we found that the electrostatic effect can be effectively minimized using two simple methods: (*A*) applying a DC voltage during a PFM measurement and (*B*) using a relatively stiff AFM cantilever. Further, we quantified the impact of the electrostatic effect on the PFM amplitude and the dependence of the effect on the stiffness of a cantilever.

## Results and Discussion

**Figure 1:**
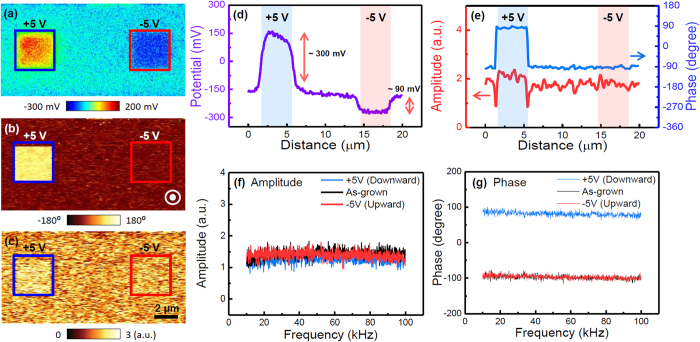
(**a**) Surface potential, (**b**) PFM phase, and (**c**) PFM amplitude of the PZT thin films. Positive and negative poles were applied to a 3 μm × 3 μm area, with a voltage of ± 5 V, using a conductive AFM tip with a spring constant of ~3 N/m. Horizontal area profiles of (**d**) the surface potential and (**e**) the PFM phase and amplitude. (**f**) PFM amplitude and (**g**) phase vs. the AC modulation frequency, performed on the poled and as-grown states. The frequency sweep was performed at a single location.

To examine a general PFM response (*i.e*., the amplitude and phase difference) for different polarization directions, we first acquired the PFM amplitude and phase images for a (001)-oriented epitaxial PZT thin film with uniform upward polarization, using an AFM tip with a spring constant of ~3 N/m (hereafter referred to as “3 N/m cantilever”). Both positive and negative voltages (+5 V and −5 V) were applied to the AFM tip for manipulating polarization directions (see Figure S1). Whereas the application of the positive voltage induced downward polarization switching (Fig. 1(b) and (c)), the application of the negative voltage did not induced polarization switching because the as-grown state of the prepared sample has upward polarization. In addition to the polarization switching, voltage application can inject charges onto the sample surface, which yields an additional local electrostatic effect between the AFM tip and sample surface by changing surface charge state, affecting the PFM response^5^,^16^. Thus, to investigate change of the surface charge state depending on the application of voltage, we performed KPFM measurements. An increase and decrease in the surface potential, compared to that of the as-grown state, were clearly observed after the application of the positive and negative voltages, as shown in Fig. 1(a) and (d). Compared to the as-grown state (around −170 mV), the surface potential increased up to ~300 mV and decreased down to ~90 mV in the positively and negatively poled regions, respectively. The decreased (increased) surface potential of the negatively (positively) poled region originates dominantly from the negative (positive) charge injection by application of negative (positive) voltage. The details can be found elsewhere^17^. We note that the asymmetry in the amount of the injected charge (comparing the positively and negatively poled regions) might be attributed to the asymmetric Schottky barrier between the AFM tip and the sample surface and/or to the Coulomb interaction between the negative surface potential of the as-grown state and the negative injected charges^15^,^16^. Despite the change of the surface charge state, the PFM phase and amplitude images in Fig. 1(b) and (c) reveal a clear 180° phase difference with similar amplitudes (slightly different, but within the error range) between oppositely poled regions. Moreover, a similar trend was also clearly observed regardless of the AC modulation frequency (Fig. 1(f) and (g)). We note that, in this study, frequency-dependent behavior was observed because the electrostatic effect could affect the frequency-dependent PFM response^8^. Therefore, we speculate that the PFM response was not significantly affected by the electrostatic effect induced by surface potentials of a few hundreds of millivolts (the surface potential difference between oppositely poled regions was ~390 mV).

**Figure 2:**
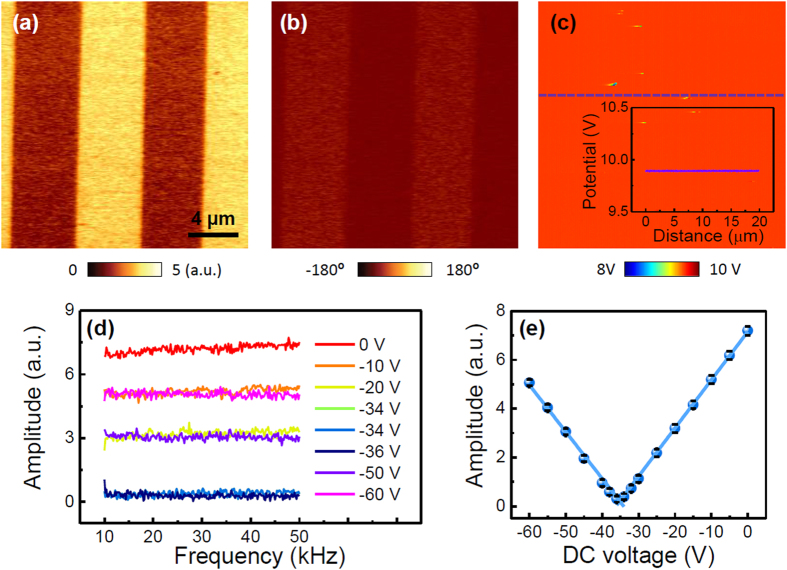
(**a**) PFM amplitude, (**b**) PFM phase, and (**c**) surface potential of PPLN obtained using the 3 N/m cantilever. In panel (c), the inset shows the line surface potential profile of the dotted line in the main plot of panel (c). (**d**) PFM amplitude vs. the AC modulation frequency in the 10–50 kHz range, and (**e**) averaged PFM amplitude vs. the DC voltage applied to the bottom electrode. The data in panels (d,e) were measured at one location in the downward-polarized regions by applying the voltage of 2 *V_ac_*. Bright blue solid lines were obtained from the least squares linear fit.

In contrast to the observed PFM response in the PZT thin films, a distorted PFM response, *e.g*., low phase difference, was observed for PPLN when the same PFM measurements were performed using the 3 N/m cantilever, as shown in Fig. 2(a) and (b). Even though it is well known that there are periodic upward and downward domains along the PPLN surface, the obtained results show different amplitudes with a very low phase difference of ~10° between opposite domains, consistent with the results reported by Proksch *et al*.^18^. Such a distorted PFM response in the case of PPLN can be attributed to the non-stoichiometry of domains and instrumental issues of AFM-based optical beam deflection (OBD) systems^18^,^19^. However, since we already confirmed that a 180° phase difference between opposite domains can be observed in our AFM OBD systems as shown in Fig. 1, instrumental issues can be ruled out as a possible explanation. That is, the observed results are expected to reflect sample-related issues. Hence, we measured the surface potential to determine the presence of the electrostatic effect between the AFM tip/cantilever and the sample surface, because this effect can distort the PFM response^6^. Figure 2(c) shows the measured surface potential for PPLN, revealing a much higher potential compared to the PZT thin film. In fact, the surface potential is often incorrectly measured, probably owing to a very high surface potential with respect to the instrumentation measurement limit ( ± 10 V) in the case of PPLN (Figure S2). This implies a considerable electrostatic effect between the AFM tip/cantilever and the PPLN surface, induced by a very high surface potential, which can significantly distort the measured PFM response.

In general, the first harmonic component of the electrostatic force (*F*_*ω*_) in the AFM system is expressed as follows^20^:


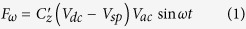


where 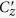, *V*_*dc*_, *V*_*ac*_, *V*_*sp*_, *ω* and *t* are the capacitance derivative along the *z* axis, the DC voltage, the AC voltage, the surface potential of the sample with respect to the AFM tip, the frequency of the AC voltage and time, respectively. In principle, the surface potential can be measured by nullifying the first harmonic component of the electrostatic force by externally applying a DC voltage in a non-contact KPFM measurement. In the contact mode case, the surface potential is obtained in a similar manner. This approach is referred to as contact KPFM, and is similar to dynamic contact electrostatic force microscopy^5^,^21^. Under this condition, it can be expected that the surface potential, which is registered by the AFM tip/cantilever, is simply modulated by externally applying a DC voltage with a magnitude different from that of *V*_*sp*_. Indeed, a change in the surface potential, depending on the magnitude of the applied external DC voltage, was experimentally observed using non-contact KPFM (Figure S3). We note that since the DC feedback voltage for nullifying the surface potential was applied to the AFM tip in the non-contact KPFM measurements, an additional external DC voltage was applied to the bottom electrode to avoid coupling with the DC feedback voltage. Thus, although we were not able to acquire the exact surface potential of PPLN owing to the measuring instrument’s limitation of KPFM ( ± 10 V), we conclude that PPLN has a positive surface potential, as shown in Fig. 2(c). Accordingly, we monitored the PFM amplitude by applying a negative voltage to the bottom electrode, *i.e*., by applying a positive voltage to the AFM tip, during the PFM measurement. In addition, to demonstrate that this approach does not significantly depend on the AC modulation frequency, we also monitored the frequency dependence of the PFM amplitude. Figure 2(d) shows the plot of PFM amplitude vs. the AC modulation frequency, for different applied external DC voltages. As the applied DC voltage increased, the PFM amplitude progressively decreased and then increased, regardless of the AC modulation frequency. To demonstrate this, the averaged PFM amplitude which reveals a clear *V*-shape is shown in Fig. 2(e). The PFM amplitude at 0 *V*_*dc*_ is 7 times larger than that at the vertex, *i.e*., around −34 *V*_*dc*_. That is, the electrostatic effect induced by the surface potential clearly affects the PFM response, which can be clearly reduced and modulated by applying an external DC voltage. In this case, since the negative voltage was applied to the bottom electrode, the surface potential of PPLN could be ~34 V, *i.e*., exhibiting an opposite vertex polarity. However, the measured surface potential varied, which might be related to the non-uniform surface state of PPLN that may be caused by the screen charges and/or humidity (Figure S2)^22^,^23^. It is worth mentioning that the PFM amplitude slightly increases with an increase in the AC modulation frequency at 0 *V*_*dc*_ (red trace in Fig. 2(d)). A similar frequency dependence of PPLN was reported by Proksch *et al*.^18^ This behavior might be also attributed to a strong electrostatic effect, because the frequency dependence was significantly attenuated after the electrostatic effect was reduced by applying a negative DC voltage during the frequency sweep (other traces in Fig. 2(d)).

**Figure 3:**
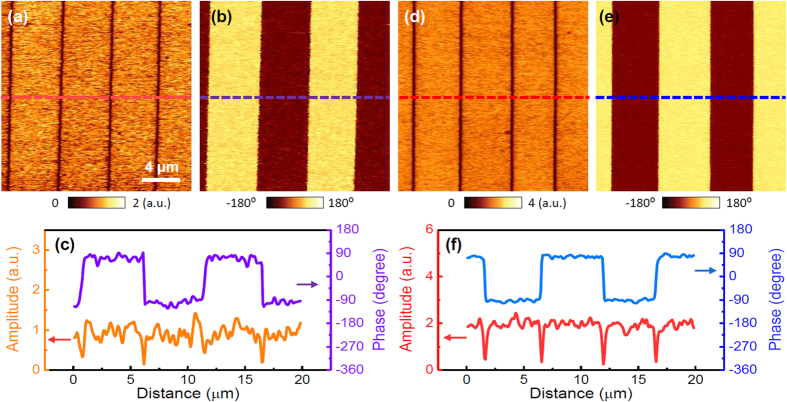
(**a**) PFM amplitude and (**b**) phase images of PPLN obtained during the application of −22 *V*_*dc*_ to the bottom electrode via the 3 N/m cantilever. (**c**) PFM amplitude and (**d**) phase images of PPLN in the as-grown state, obtained using the 42 N/m cantilever. (**e**,**f**) Line profiles of the PFM amplitude and phase images corresponding to the dotted lines in panels (a–d), respectively.

Using the results in Fig. 2, we acquired PFM images as a negative DC voltage in the vicinity of the vertex was applied to the bottom electrode. Figure 3(a) and (b) show the PFM amplitude and phase images of PPLN, obtained for the applied voltage of −22 V. As mentioned above, since the surface potential of PPLN slightly differs depending on the measured region, the DC voltage at the vertex can vary. Interestingly, similar amplitudes with a clear phase difference of 180° between opposite domains were clearly observed for PPLN, which are shown in Fig. 3(a–c). These observations suggest that reasonable-quality PFM images can be obtained by applying an external DC voltage, because it reduces the electrostatic effect. It is noted that this approach can be limited if surface potential is higher than coercive voltages because external DC voltage for nullifying electrostatic effect can induce polarization switching. However, in many cases, as-grown surface potential is much lower than typical coercive voltages of ferroelectric thin films. (see the coercive voltage of the PZT thin film in Figure S1)^17^,^24^,^25^ Thus, this approach is also applicable for the thin films.

It is well known that the first harmonic response of a cantilever, (*PR*)_*ω*,_ in the PFM can be expressed as follows:^15^


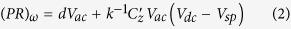


where *d* and *k* are the piezoelectric coefficient and the spring constant of the cantilever, respectively. The second term in the above equation is related to the electrostatic force, similar to equation (1). This implies that the impact of the surface potential, *i.e*., the electrostatic force, can be reduced by either: (*A*) applying an external DC voltage as mentioned above (*i.e*., *V*_*dc*_ = *V*_*sp*_), or (*B*) by using a stiff AFM cantilever (*i.e*., *k*^*−*1^ ≪ 1), because the second term is proportional to the difference between the magnitude of the applied DC voltage and the surface potential and inversely proportional to the spring constant of the cantilever. In fact, it is theoretically and experimentally well known that the electrostatic effect can be alleviated using a relatively stiff cantilever^5^,^26^,^27^. Since method *A* was already demonstrated in Fig. 2 and Fig. 3(a) and (b), we performed additional PFM measurements using an AFM tip with a spring constant of ~42 N/m (hereafter referred to as “42 N/m cantilever”) to demonstrate method *B*. Interestingly, unlike the PFM images obtained using the 3 N/m cantilever, clear PFM images were obtained using the 42 N/m cantilever, and the results are shown in Fig. 3(d–f). These results, too, suggest that the electrostatic effect between the AFM tip/cantilever and the PPLN surface is a major mechanism underlying the PFM response distortion in PPLN. Note that this trend was observed regardless of the modulation frequency (Figure S4).

In addition to the PFM imaging, and because the measured PFM amplitude also decreased significantly when the electrostatic effect was alleviated as shown in Fig. 2(e), we further investigated its effect on quantitative PFM measurements by performing AC voltage sweeps^28^. In these AC voltage sweeps, we measured the PFM amplitude induced by gradually increasing the AC voltage at a fixed frequency, and plotted the results vs. the AC voltage^28^. In these plots, the slope corresponds to the piezoelectric coefficient (*i.e*., *d*) if the measured PFM amplitude is determined only by the piezoresponse (the ideal case). However, owing to the electrostatic effect, the slope cannot be considered to represent an intrinsic piezoelectric coefficient. In other words, an effective slope (defined here as an effective piezoelectric coefficient, *d*_*eff*_, which includes both piezoelectric and electrostatic contributions) of the PFM amplitude vs. the AC voltage can be expressed by considering the electrostatic contributions, as follows:


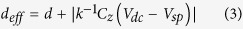


**Figure 4:**
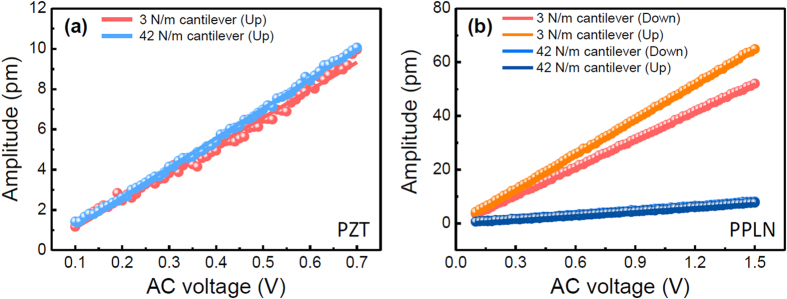
Calibrated PFM amplitude vs. the AC voltage, for (**a**) the PZT thin film and (**b**) PPLN. The ‘up’ and ‘down’ labels in the figure legends indicate the polarization directions. The solid lines are the least squares linear fits. AC voltage sweeps were averaged over 16 sets of data points.

AC voltage sweeps were performed for both samples and the obtained slopes (*i.e. d*_*eff*_) were calibrated by performing a general calibration procedure based on the static force-distance curve, as shown in Fig. 4^29^. It is noted that the dynamic calibration method recently suggested by Balke *et al*.^26^ may provide better accuracy on the quantified values compared to the static force-distance curve. However, since the static calibration method based on the force-distance curve has been widely used and, further, we focus on the observation of the contribution of the electrostatic effect, the present static calibration method can be sufficient to show quantitative electrostatic contribution. In the case of the PZT thin film, the effective piezoelectric coefficients obtained using the 3 N/m and 42 N/m cantilevers were nearly the same (13.7 pm/V and 14.7 pm/V, respectively). As mentioned above, the electrostatic effect induced by the surface potential was not significant in the PZT thin film, owing to the relatively lower surface potential (around −170 mV); hence, the effective piezoelectric coefficient could be considered to be the intrinsic piezoelectric coefficient and was nearly the same, regardless of the cantilever stiffness. Note that a cantilever with a spring constant lower than 3 N/m may yield a stronger influence of the electrostatic effect on the observed results.

On the other hand, the obtained slopes (*i.e*., the effective piezoelectric coefficients) for PPLN have distinctly different values, depending on the cantilever stiffness and polarization direction, and these results are summarized in Table S1. For the 3 N/m cantilever, the effective piezoelectric coefficients were significantly larger than those for the 42 N/m cantilever in both opposite domains and there were also significant differences between the upward and downward polarization domains. Note that a large difference between the effective piezoelectric coefficients of opposite domains might be related to the difference between local surface potentials^26^. However, for the 42 N/m cantilever, the effective piezoelectric coefficients for both domains of PPLN were ~5 pm/V. This value is of the same order of magnitude as the previously reported value of ~8 pm/V (albeit slightly lower)^18^.

**Figure 5:**
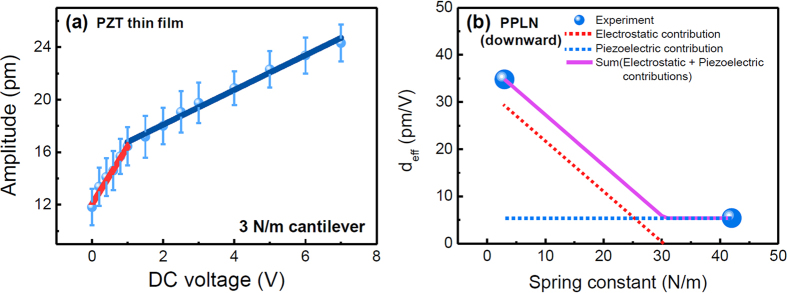
(**a**) Averaged PFM amplitude vs. the DC voltage applied to the bottom electrode in the PZT thin film. The PFM amplitude was measured at one location in the upward polarized region, for frequencies in the 10–50 kHz range. The above result was obtained using the 3 N/m cantilever. The red and blue solid lines are the least squares fits. (**b**) The effective piezoelectric coefficient obtained for the downward domain of PPLN, vs. the spring constant of a cantilever. The red dotted line in panel (**b**) shows the electrostatic contribution to the effective piezoelectric coefficient obtained from equation (3), using the experimentally obtained 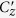 in panel (a). The blue dotted line represents the intrinsic piezoelectric contribution to the effective piezoelectric coefficient, obtained for the experimental data in Fig. 4(b). The purple solid line is the sum of both the electrostatic and piezoelectric contributions.

Because the electrostatic effect significantly affected the measured PFM response, we quantified its contribution using the same cantilever, intentionally increasing the surface potential by applying an external DC voltage to the PZT thin film. Figure 5(a) shows the obtained PFM amplitude as a function of the DC voltage applied to the bottom electrode. Since the as-grown state of the PZT thin film shows upward polarization, we only applied a positive voltage, to avoid polarization switching. The PFM amplitude increased with increasing the applied DC voltage. This implies that the electrostatic effect contributes to the PFM amplitude for surface potentials as small as a few hundreds of millivolts. However, it is worth mentioning that such an influence of the electrostatic effect might be difficult to detect for voltages up to ~0.6 V, especially for the PFM images, owing to overlapping error bars. In other words, even though the electrostatic effect is significant up to ~0.6 V, it is difficult to clearly distinguish its contribution from the PFM response, owing to the deviation of the PFM response itself, which was indeed observed in Fig. 1^30^. Interestingly, the two linear trends exhibited crossover at 1 V. Below 1 V, the PFM amplitude increased relatively steeply compared to voltages >1 V. This behavior was repeatedly observed at different locations (Figure S5). The slopes of the first and second linear segments, obtained from least squares linear fits, were 4.6 pm/V and 1.4 pm/V, respectively. That is, in these two regions, the electrostatic effect increased the PFM amplitude by as much as 4.6 pm and 1.4 pm per 1 V of the DC voltage. For relatively low voltages (under 1 V), the injected negative charges also contributed to the PFM response as a local electrostatic effect. That is, both locally injected charges and non-locally induced surface potential resulted in the electrostatic effect that eventually affected the observed PFM amplitude for voltages under 1 V. However, for voltages above 1 V, the non-local electrostatic effect became dominant, because the local electrostatic effect induced by the injected charges becomes insignificant as the non-local surface potential increased. In fact, a change in the surface potential induced by the negative injected charges was only around −90 mV, which was not significant, considering the application of −5 V to the AFM tip (Fig. 1(d)). Note that, owing to a fast charge relaxation that cannot be properly detected by the KPFM measurement, the actual surface potential can be slightly higher than the observed value. On the other hand, it is worth mentioning that this local electrostatic effect could be correlated with nonlinear behavior at a low AC voltage regime (<1 V) during an AC voltage sweep reported by Lei *et al*.^31^.

Furthermore, because a higher stiffness of a cantilever can reduce the electrostatic effect’s contribution to the observed PFM response^32^, we characterized stiffness-dependent behavior of the effective piezoelectric coefficient on the downward polarization domains of PPLN. As shown in Fig. 4, whereas the effective piezoelectric coefficient obtained using the 3 N/m cantilever was strongly influenced by the electrostatic effect, the electrostatic effect for the 42 N/m cantilever was negligibly small. Thus, to explore the stiffness dependence of the effective piezoelectric coefficient, we assumed the following: (1) the electrostatic contribution is negligible for stiffness above a certain threshold, (2) the effective piezoelectric coefficient measured using the 42 N/m cantilever is the intrinsic piezoelectric coefficient of PPLN (blue dotted line in Fig. 5(b), (3) the geometry effect associated with the different cantilevers is negligible (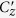 is constant regardless of the cantilever type). Using the above assumptions, the electrostatic contribution to the effective piezoelectric coefficient was calculated simply by obtaining 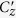 by fitting equation (2) to the second line (blue line) in Fig. 5(a), because *k* and *V*_*sp*_ were given as 3 N/m and 34 V, respectively. The calculated electrostatic contribution is shown by the red dotted line in Fig. 5(b). We note that 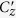 was obtained from the PZT thin film owing to its relatively uniform surface compared to that of PPLN (Fig. 2). By combining both the electrostatic (red dotted lines in Fig. 5(b)) and the intrinsic piezoelectric (blue dotted line in Fig. 5(b)) contributions to the PFM response, the overall effective piezoelectric coefficient as a function of the spring constant was reconstructed and is shown by the purple solid line in Fig. 5(b). The reconstructed result clearly shows that the effective piezoelectric coefficient decreases with the increase in the stiffness of the cantilever and remains constant above a certain spring constant (here, 30.5 N/m), corresponding to the intrinsic piezoelectric coefficient. Note that the specific value of the spring constant can differ depending on the surface potential of the measured sample. Nonetheless, from the obtained results, a reduction of 1 N/m in the spring constant increases the contribution of the electrostatic effect to the PFM response by 0.75 pm per unit AC voltage.

## Conclusion

In conclusion, to explore the contribution of the electrostatic effect to the EM response as well as obtain the electrostatic-free EM response, we quantitatively investigated the impact of the electrostatic effect on PFM (as a model AFM system), using two model samples – the PZT thin film and PPLN. We found that a strong electrostatic effect owing to the higher surface potential can distort the PFM amplitude and phase contrast in the case of PPLN. However, the impact of this effect can be effectively minimized by employing two simple methods: (*A*) applying a DC voltage for nullifying the surface potential and (*B*) using a relatively stiff AFM cantilever compared to the surface potential. In addition, we quantitatively investigated the impact of the electrostatic effect on the PFM amplitude. An increase of 1 V in the surface potential, i.e., an increased electrostatic contribution, increased the PFM amplitude by 1.4 pm. Moreover, the PFM amplitude increased by as much as 0.75 pm per unit AC voltage after decreasing the spring constant by 1 N/m. These observations imply that the electrostatic effect can directly affect the measured EM response and hinder accessing the electrostatic-free EM response. Although the impact of the electrostatic effect was observed for the PFM, we anticipate that these results will also be applicable to other AFM-based measurements, regardless of contact and non-contact modes, owing to the concurrent nature of the electrostatic effect. Thus, these observations provide basic guidelines for accurate measurement and interpretation of AFM-based quantities, when there is a strong electrostatic effect between the AFM tip/cantilever and sample surface.

## Methods

### Materials

A 90-nm-thick (001)-oriented epitaxial PZT thin film was prepared by pulsed laser deposition on a SrRuO_3_/SrTiO_3_ substrate at T = 600 °C and P_O2_ = 0.15 Torr^33^ and PPLN was purchased from the Asylum Research.

### Measurements

We used a commercial AFM (NX10, Park Systems) connected with a lock-in amplifier (SR830, Stanford Research) for the PFM measurements. Two types of Pt/Ir coated conductive AFM tips were used, with spring constants of 3 N/m (Multi75E-G, BudgetSensors) and 42 N/m (PPP-NCHPt, Nanosensors). A 0.5 V (2 V) AC modulation voltage at 17 kHz was applied to the AFM tips for PFM imaging of the PZT thin film (PPLN). The frequency dependence of the PFM response was observed by applying *V*_*ac*_ of 0.5 V (2 V) to the PZT thin film (PPLN), and AC amplitude sweeps were performed by applying voltages in the 0.1–0.7 V range (0.1–1.5 V range) to the PZT thin film (PPLN), at a fixed frequency of 17 kHz, using custom LabVIEW software. To measure the surface potential, we used amplitude-modulated KPFM. The AC modulation voltage of 2 V at 17 kHz and the DC feedback voltage were applied to the AFM tip and a two-scan mode was used. In the second scan, the tip-sample distance of 50 nm was increased to reduce topographical crosstalk^34^.

## Additional Information

**How to cite this article**: Kim, S. *et al*. Electrostatic-free piezoresponse force microscopy. *Sci. Rep.* **7**, 41657; doi: 10.1038/srep41657 (2017).

**Publisher's note:** Springer Nature remains neutral with regard to jurisdictional claims in published maps and institutional affiliations.

## Supplementary Material

Supplementary Information
