# Supplementary material for: Electrostatic-free piezoresponse force microscopy
Source: Sci Rep. 2017 Jan 31;7:41657. doi: 10.1038/srep41657 (PMC5282565; doi:10.1038/srep41657)
Supplement: Supplementary Information [file srep41657-s1.doc]

**Supplementary Information**

**Electrostatic-free piezoresponse force microscopy**

*Sungho Kim^a^, Daehee Seol^a^, Xiaoli Lu^b,*^, Marin Alexe^c^, and Yunseok Kim^a,*^*

^a^School of Advanced Materials Science and Engineering, Sungkyunkwan University (SKKU), Suwon, 16419, Republic of Korea

^b^The State Key Discipline Laboratory of Wide Band Gap Semiconductor Technology, Xidian University, Xi'an, Shaanxi 710071, China

^c^Department of Physics, University of Warwick, Coventry CV4 7AL, United Kingdom

*Address correspondence to: xllu@xidian.edu.cn and yunseokkim@skku.edu


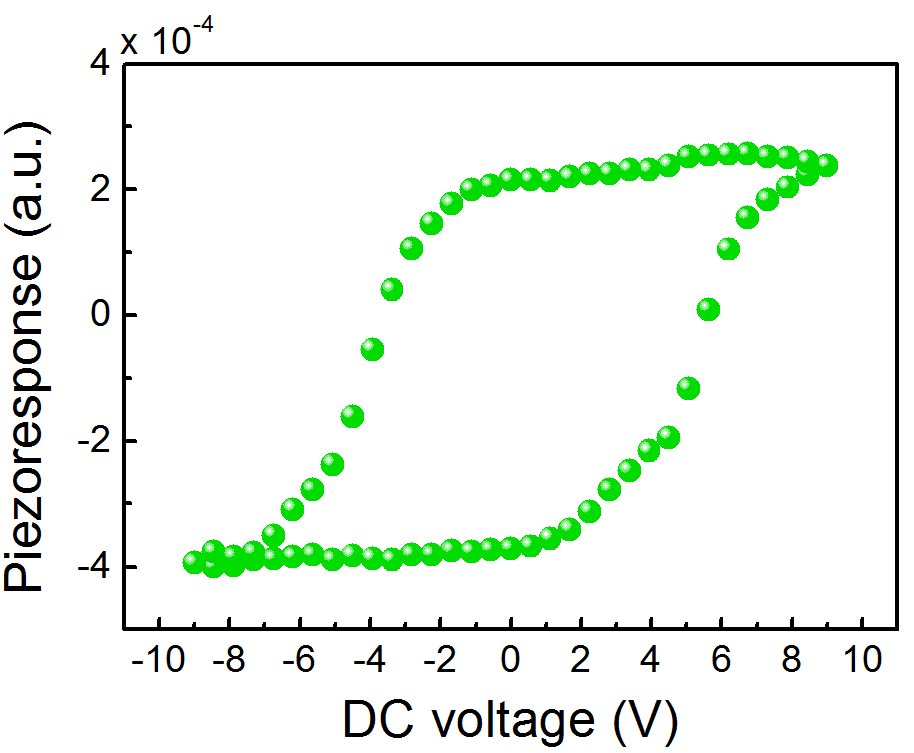


Figure S1. Piezoresponse hysteresis loop of the PZT thin film. This result was obtained from average of 16 measurements.


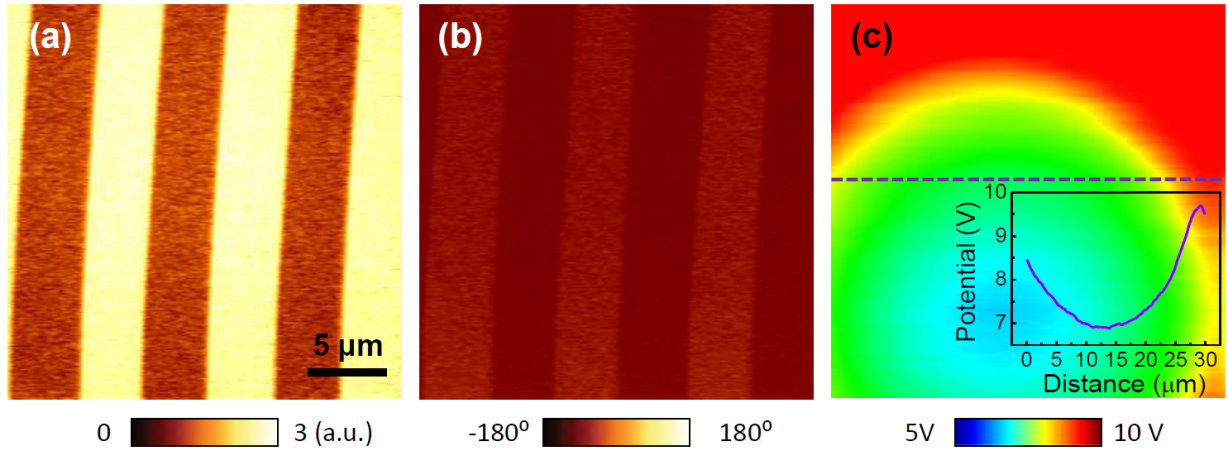


Figure S2. (a) PFM amplitude and (b) phase images of PPLN, and (c) surface potential of PPLN, obtained using the 3 N/m cantilever. The inset in panel (c) shows the line profile of the surface potential.


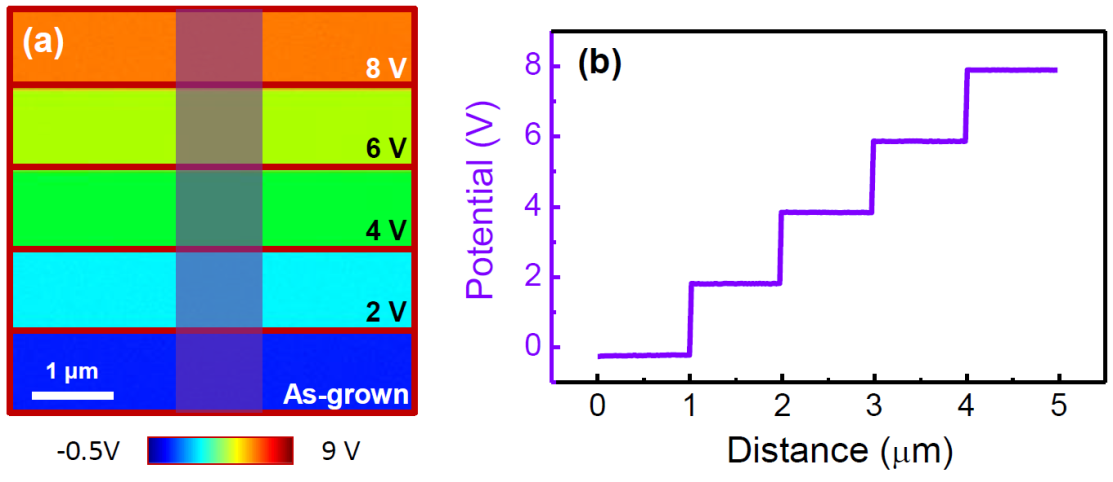


**Figure S3.** (a) Surface potential of the PZT thin film during application of DC voltage to the bottom electrode. (b) The corresponding vertical area profile of the surface potential.


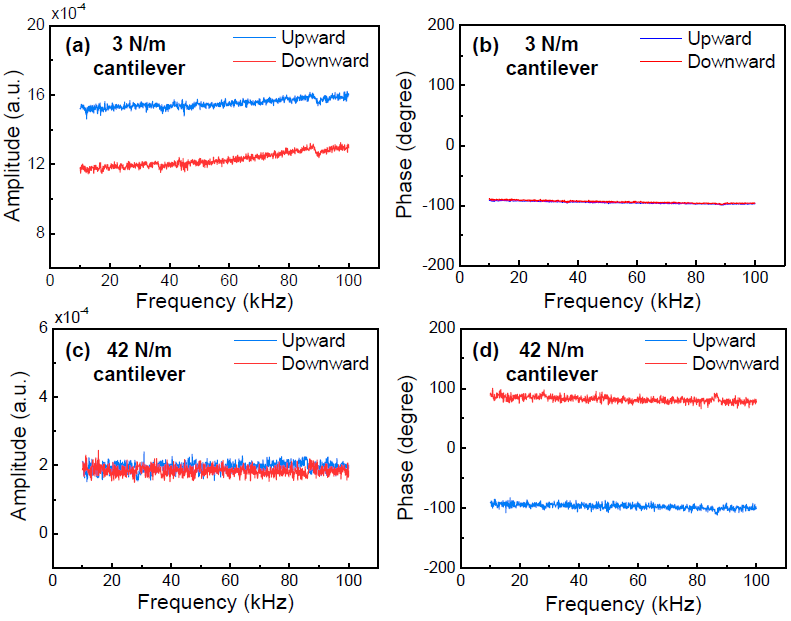


**Figure S4.** (a), (c) PFM amplitude and (b), (d) phase vs. AC modulation frequency measured for the upward and downward polarizations of PPLN. Note that (a), (b) and (c), (d) were obtained using 3 N/m and 42 N/m cantilevers, respectively. The above results were obtained at a single location.

For both 3 N/m and 42 N/m cantilevers, the results were similar to those shown in the PFM images in Figure. 2, regardless of the AC modulation frequency. A slight increase in the PFM amplitude with increasing frequency and different amplitudes across opposite domains was observed when the 3 N/m cantilever was used. In addition, there was no clear phase difference between the upward and downward domains, regardless of the AC modulation frequency. However, by using the 42 N/m cantilever, similar PFM amplitudes and a clear 180° phase difference between opposite domains were observed. Furthermore, the frequency dependence of the PFM amplitude vanished. These results clearly indicate that appropriate PFM responses can be observed by reducing the electrostatic effect by using a stiff cantilever, regardless of the AC modulation frequency.


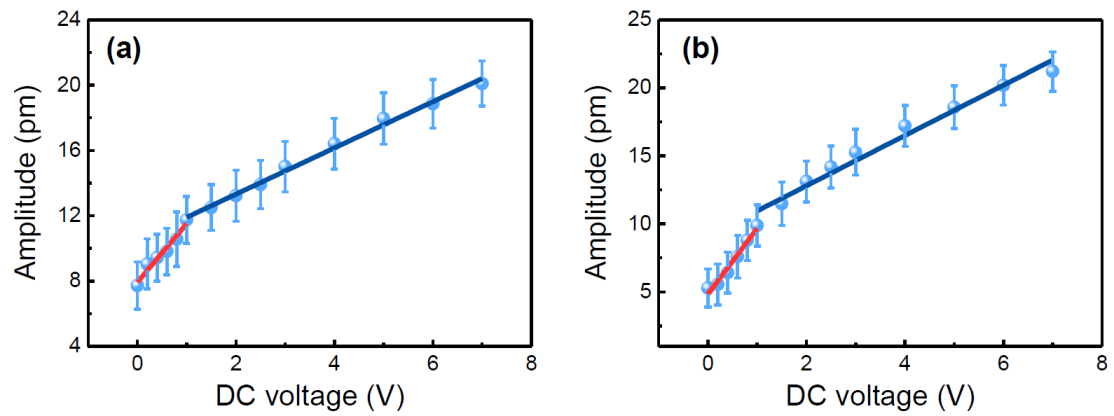


Figure S5. (a, b) Averaged PFM amplitude vs. the DC voltage applied to the bottom electrode in the PZT thin film. The PFM amplitude was measured at a single location in the upward polarized region, for frequencies in the 10–50 kHz range. The above results were obtained using the 3 N/m cantilever. The red and blue solid lines are the least squares linear fits. Note that the measured amplitude was normalized by the AC voltage magnitude.

**Table S1.** Effective piezoelectric coefficients of the PFM amplitude with respect to magnitude of AC voltage of the PZT thin film and PPLN, extracted from the linear fits in Figure. 4. ‘Up’ and ‘down’ denote the polarization directions.

|  | Spring constant (N/m) | PZT | PPLN | |
| --- | --- | --- | --- | --- |
|  |  | Up | Down | Up |
| *d_eff_* (pm/V) *(standard deviation)* | 3 | 13.7 (±2.19) | 34.8 (±1.04) | 43.3 (±0.48) |
|  | 42 | 14.7 (±2.43) | 5.4 (±0.20) | 5.0 (±0.34) |
